# Young transposable elements rewired gene regulatory networks in human and chimpanzee hippocampal intermediate progenitors

**DOI:** 10.1242/dev.200413

**Published:** 2022-10-04

**Authors:** Sruti Patoori, Samantha M. Barnada, Christopher Large, John I. Murray, Marco Trizzino

**Affiliations:** ^1^ Department of Biochemistry and Molecular Biology, Sidney Kimmel Medical College, Thomas Jefferson University, Philadelphia, PA 19107, USA; ^2^ Department of Genetics, Perelman School of Medicine, University of Pennsylvania, Philadelphia, PA 19104, USA

**Keywords:** Hippocampal development, Transposable elements, Induced pluripotent stem cells, Intermediate progenitors, TBR2, SINE-Vntr-Alus

## Abstract

The hippocampus is associated with essential brain functions, such as learning and memory. Human hippocampal volume is significantly greater than expected compared with that of non-human apes, suggesting a recent expansion. Intermediate progenitors, which are able to undergo multiple rounds of proliferative division before a final neurogenic division, may have played a role in evolutionary hippocampal expansion. To investigate the evolution of gene regulatory networks underpinning hippocampal neurogenesis in apes, we leveraged the differentiation of human and chimpanzee induced pluripotent stem cells into TBR2 (or EOMES)-positive hippocampal intermediate progenitor cells (hpIPCs). We found that the gene networks active in hpIPCs are significantly different between humans and chimpanzees, with ∼2500 genes being differentially expressed. We demonstrate that species-specific transposon-derived enhancers contribute to these transcriptomic differences. Young transposons, predominantly endogenous retroviruses and SINE-Vntr-Alus (SVAs), were co-opted as enhancers in a species-specific manner. Human-specific SVAs provided substrates for thousands of novel TBR2-binding sites, and CRISPR-mediated repression of these SVAs attenuated the expression of ∼25% of the genes that are upregulated in human intermediate progenitors relative to the same cell population in the chimpanzee.

## Teaser

**Highlighted Article:** Evolution of human and chimpanzee hippocampal development was mediated by the co-option of young retrotransposons into species-specific enhancers.

## INTRODUCTION

The hippocampus is associated with many traits relevant in the context of human evolution. These include traits such as tool use and language, which require social cognition and learning, as well as spatial memory, navigation and episodic memory (Burgess et al., 2002; Eichenbaum, 2017a,b; Squire, 1992; Tomasello and Herrmann, 2010). This region of the brain is also greatly affected by Alzheimer's Disease (AD), a neurodegenerative disorder characterized by cell death, plaques and tangles of misfolded proteins, and cognitive decline (Duyckaerts et al., 2009). It has been hypothesized that the cognitive AD phenotype is uniquely human and that non-human primates, including chimpanzees, do not exhibit AD-related dementia (Edler et al., 2017; Finch and Austad, 2015; Walker and Jucker, 2017). If humans are uniquely susceptible to AD, it is crucial to understand how the human hippocampus differs from that of our closest biological relatives, the chimpanzees.

Human hippocampal volume is 50% greater than expected compared with the hippocampal volumes of non-human apes, possibly indicating a recent hippocampal expansion specific to the human lineage (Barger et al., 2014). However, the evolution of the human hippocampus and the developmental mechanisms driving the human-specific volume increase have not yet been thoroughly studied.

Recent studies have suggested that evolutionary changes to neuronal progenitors may have had an impact on cortical volume in primates by increasing proliferative potential (Martínez-Cerdeño et al., 2006; Rétaux et al., 2013; Florio and Huttner, 2014). A specific class of neuronal progenitors known as intermediate progenitor cells (IPCs) or ‘transit-amplifying cells’ are able to undergo multiple rounds of proliferative division before a final neurogenic division (Englund, 2005; Arnold et al., 2008; Pontious et al., 2008; Hevner, 2019). These cells express the neurodevelopmental transcription factor TBR2 (EOMES) and are found in the sub-ventricular zone of the developing neocortex and hippocampus (Bulfone et al., 1999; Kimura et al., 1999; Englund, 2005; Cipriani et al., 2016). Genetic ablation of TBR2 in these progenitors results in reduced cortical thickness in mice (Sessa et al., 2008), abnormal cortical cell differentiation (Mihalas et al., 2016) and impaired neurogenesis in the hippocampal formation (Hodge et al., 2012). As these IPCs are hypothesized to play a role in neocortical expansion, they may also be involved in the lineage-specific hippocampal expansion seen in humans.

Many of the differences between humans and chimpanzees are due to diverging gene regulatory sequences (Agoglia et al., 2021; Enard et al., 2002; Gokhman et al., 2021; King and Wilson, 1975; Wray, 2007). A recent study comparing gene expression in adult human, chimpanzee and macaque brain regions identified several genes specifically upregulated in the human hippocampus (Sousa et al., 2017). However, transcriptomic differences between primate species during specific time points of hippocampal development have not been investigated.

As samples of developing human and chimpanzee brain tissue are extremely limited, induced pluripotent stem cells (iPSCs) are an ideal system to conduct comparative studies of human and chimpanzee hippocampal development. Previous studies have employed iPSC-derived cortical organoids and iPSC-derived neuronal progenitor cells (NPCs) from human and chimpanzee for comparative and developmental genomic purposes (Marchetto et al., 2019; Mora-Bermúdez et al., 2016). Here, we leverage human and chimpanzee iPSC-derived hippocampal progenitors as models for comparative developmental and genomic studies with the goal of identifying species-specific differences in gene regulation during hippocampal development.

Several papers have recently demonstrated that transposable elements (TEs) can alter existing regulatory elements or generate entirely novel ones, as well as expand in a species- or lineage-specific manner (Sundaram et al., 2014; Playfoot et al., 2021; Okhovat et al., 2020; Marnetto et al., 2018; reviewed by Sundaram and Wysocka, 2020). Species-specific TE expansion and co-option into gene regulatory networks have been demonstrated as a mechanism for evolutionary change (Chuong et al., 2016; Fuentes et al., 2018; Jacques et al., 2013; Lynch et al., 2011, 2015; Miao et al., 2020; Mika et al., 2021; Pontis et al., 2019; Trizzino et al., 2017). Endogenous retroviruses (ERVs) and SINE-Vntr-Alus (SVAs) are among the TE families more frequently associated with gene regulatory activity in the human genome (Chuong et al., 2016; Fuentes et al., 2018; Pontis et al., 2019; Trizzino et al., 2017, 2018). SVAs encompass six subfamilies, denoted as SVA-A through SVA-F. Of the ∼3000 SVA copies in the human genome, nearly half are human specific, including those belonging to the SVA-E and SVA-F subfamilies (Quinn and Bubb, 2014; Wang et al., 2005). The remaining half are also found in other great apes. SVAs are still replication competent and thus able to transpose in the human genome. ERVs are retrotransposons belonging to the long terminal repeat (LTR) group. They are remnants of past retroviral infection events and make up ∼8% of the human genome (Tokuyama et al., 2018). Both SVAs and ERVs were recently found to be enriched within the sequences of active cis-regulatory elements (enhancers and promoters) in hippocampal tissue compared with other human brain regions in which they are predominantly repressed (Trizzino et al., 2018). Therefore, we hypothesize that species-specific ERV and SVA transposon activity may influence the gene regulatory networks necessary for human and chimpanzee hippocampal development.

Given the key function that IPCs played in the evolution of the primate brain (Florio and Huttner, 2014; Martínez-Cerdeño et al., 2006), we sought to identify molecular differences between iPSC-derived human and chimpanzee hippocampal intermediate progenitor cells (hpIPCs) in terms of gene expression and the regulatory activity of non-coding regions. We specifically examined gene expression [by RNA sequencing (RNA-seq) and single-cell RNA-seq (scRNA-seq)], gene regulation [by assay for transposase-accessible chromatin using sequencing (ATAC-seq)] and functional TE activity via CRISPR interference.

We leveraged scRNA-seq to examine the temporal trajectory of the hpIPCs during differentiation in both species. After confirming that the hpIPC differentiated cells express the appropriate neurodevelopmental markers, we conducted a transcriptomic comparison between human and chimpanzee hpIPCs. This analysis revealed over 2500 differentially expressed genes (DEGs). We then used ATAC-seq to conduct extensive analyses of differential chromatin accessibility between human and chimpanzee hpIPCs. In both species, differentially accessible (DA) chromatin regions were more likely than expected to overlap a TE insertion. Furthermore, these regions were found to be both enriched and depleted for specific TE families. Notably, species-specific enrichment for ERV and SVA sequences within differentially accessible genomic sites correlated with species-specific changes in nearby gene expression. This is likely driven by transcription factors binding to the TE-derived regulatory sequences, as we demonstrate for TBR2 and SVA-derived enhancers. Finally, we used CRISPR interference to repress all the accessible SVAs in progenitor-like cells and demonstrated that such repression results in global changes in gene expression and affects hundreds of important neurodevelopmental genes.

This work demonstrates that two young TE families have contributed significantly to the gene regulatory differences between human and chimpanzee hippocampal development, providing insight into how the human hippocampus evolved both its unique cognitive capacity and its susceptibility to neurodegenerative disease.

## RESULTS

### An iPSC-derived model for human and chimpanzee hpIPCs

We modeled hpIPCs in humans and chimpanzees using three human and three chimpanzee iPSC lines. All six iPSC lines were validated as pluripotent in previous studies (Gallego Romero et al., 2015; Pagliaroli et al., 2021; Pashos et al., 2017; Ward et al., 2018; Yang et al., 2015; Zhang et al., 2015). For both human and chimpanzee iPSCs, we used two female cell lines and one male line.

We used a previously published method to generate hpIPCs from iPSCs (Yu et al., 2014). In this protocol, the stem cells are treated with a medium containing anticaudalizing factors and sonic hedgehog antagonists (DKK1, Noggin and SB431542) to generate forebrain progenitor cell types (Yu et al., 2014). It should be noted that the hpIPCs are distinct from a more general neuronal progenitor cell (pan-NPC) as the pan-NPC medium is supplemented only with FGF2 and B27. Moreover, the hpIPCs can be further induced to generate mature hippocampal CA3 pyramidal or dentate gyrus granule neurons (Sarkar et al., 2018; Yu et al., 2014).

**Fig. 1.**
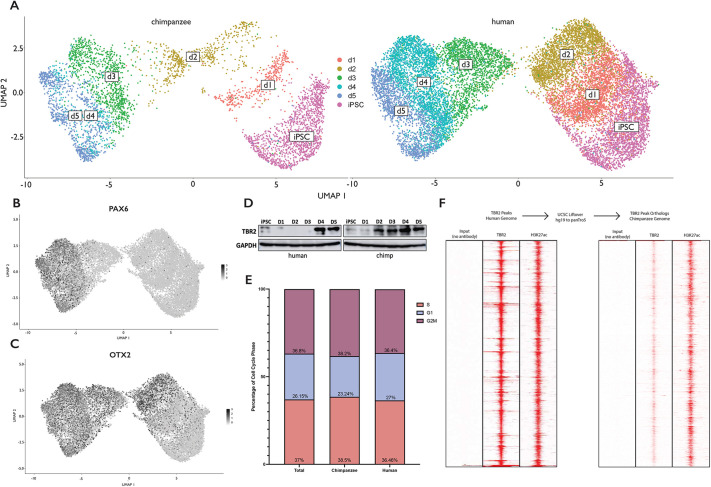
**Differentiation of human and chimpanzee iPSCs into hpIPCs.** (A) scRNA-seq data of human and chimpanzee cells from iPSCs to day (d) 5 of hpIPC differentiation, grouped by time point and split by species. Uniform manifold approximation and projection, UMAP. (B,C) *PAX6* and *OTX2* expression across the hpIPC differentiation time points. Darker gray indicates greater expression. (D) Western blotting of human and chimpanzee differentiated iPSC samples against TBR2 from day 0 to day 5 of differentiation. GAPDH is shown as the loading control. Images are representative of two replicates. (E) Quantification of cells in the S, G1 and G2/M phases of the cell cycle from scRNA-seq data. (F) Heatmaps of ChIP-seq against TBR2 and H3K27ac in day-5 hpIPCs from human and chimpanzee. Red indicates signals.

As we were specifically interested in TBR2-positive hpIPCs, we differentiated one human male and one chimpanzee male iPSC line to ensure that differentiation proceeded similarly in each species-specific cell line and we compared the transcriptomes by conducting scRNA-seq at 24 h intervals from the iPSC stage (day 0) to the hpIPC stage (day 5). We assayed a total of 18,935 cells, with 4540 chimpanzee cells and 14,395 human cells. The scRNA-seq data demonstrate that the differentiation follows a similar trajectory in both species (Fig. 1A). Despite the difference in final cell number between each species-specific cell line (Fig. S1A), all six time points overlap closely between the species (Fig. S1B,C). Chimpanzee cells from days 2 and 3 overlap more than expected with human cells from days 3 and 4, respectively, suggesting that the chimpanzee cells are further along in differentiation to hpIPCs at these time points. However, both species align closely again by day 5, which is the time point chosen for all the genomic analyses conducted in the present study.

We further leveraged the scRNA-seq data to examine the expression of known neurodevelopmental markers and saw a progressive increase in the expression of both *PAX6* and *OTX2* during the 5-day differentiation (Fig. 1B,C), which remained consistent between species (Fig. S1D,E). To investigate any noticeable changes to the cell cycle or proliferation between species, we quantified the number of cells at the G1, S and G2/M phases of the cell cycle within the scRNA-seq dataset based on a set of known cell cycle genes and did not detect significant differences (Fig. 1E).

To ensure that the differentiation resulted in hpIPCs specifically, we conducted western blotting for samples from both species-specific cell lines and confirmed that TBR2 expression in human cells was greatest at days 4 and 5 (Fig. 1D), similarly to *OTX2* and *PAX6* expression (Fig. 1B,C). In chimpanzee cells, TBR2 was expressed as early as day 2, corroborating evidence from the scRNA-seq data that chimpanzee differentiation initially proceeds faster. However, we did not see evidence that TBR2 expression in chimpanzee cells was greater than TBR2 expression at day 5 in human cells. As OTX2 labels neuronal progenitors and TBR2 and PAX6 mark the intermediate progenitor cell type (Florio and Huttner, 2014; Cipriani et al., 2016; Hevner, 2019), the data demonstrate that the differentiation was successful and comparable between species. Lastly, we conducted chromatin immunoprecipitation (ChIP) followed by sequencing (ChIP-seq) against TBR2 and the active histone mark H3K27ac to determine whether TBR2 was bound at the same genomic sites in both species (Fig. 1F). We identified 3789 TBR2-bound regions in human hpIPCs and observed that nearly all of them also exhibited H3K27ac. Upon translating the coordinates of these regions to the chimpanzee genome, we observed that the 3781 orthologous regions were bound by TBR2 in chimpanzee hpIPCs. Together, these data indicate that our iPSC-derived model is suitable to study gene regulation within TBR2-positive hpIPCs from both human and chimpanzee.

### Important neurodevelopmental genes are differentially expressed between human and chimpanzee hpIPCs

**Fig. 2.**
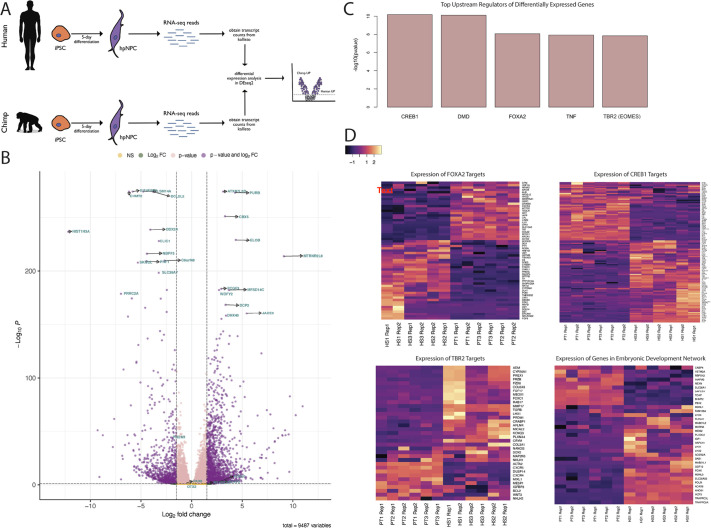
**Differential gene expression in human and chimpanzee hpIPCs.** (A) Schematic of iPSC differentiation followed by RNA-seq library generation and analysis. Hippcampal NPC, hpNPC. (B) Volcano plot depicting ‘Human UP’ genes (right) and ‘Chimp UP’ genes (left) with a log_2_[fold change (FC)] threshold of 1.5 and −1.5, respectively, and a *P*-value threshold of 0.05. (C) Top upstream regulators of the DEGs predicted by Ingenuity pathway analysis, ranked by −log_10_*P*. (D) Heatmaps depicting the expression of genes predicted to be under the control of the transcription factors CREB1, FOXA2 and TBR2 or predicted to play a role in embryonic development. The rows of the heatmaps indicate the transcript names, columns indicate the species, sample number and replicate. For example, ‘HS1 Rep 1’ indicates *Homo sapiens* sample 1 replicate 1, and ‘PT2 Rep 2’ indicates *Pan troglodytes* sample 2 replicate 2.

To investigate the differences between human and chimpanzee hpIPCs, we first aimed to characterize differential gene expression between the hpIPCs of the two species. After 5 days of treatment with the hpIPC differentiation medium, we collected cells for RNA extraction (Fig. 2A). To ensure statistical power, we conducted bulk RNA-seq on two replicates each of all six cell lines (i.e. three biological replicates and six technical replicates per species). The differentiation, harvesting and RNA processing were performed in mixed batches with samples from both species to prevent batch effects. The libraries were sequenced using an Illumina NextSeq500, generating 100 bp paired-end reads. Non-orthologous genes were omitted from the analysis and a total of 2588 genes were identified as being differentially expressed [false discovery rate (FDR)<0.05 and log_2_(fold change)>1.5 or <−1.5]. The genes with log_2_(fold change)>1.5 were more highly expressed in the human hpIPCs (‘Human UP’), whereas the genes with log_2_(fold change)<−1.5 were more highly expressed in the chimpanzee hpIPCs (‘Chimp UP’). In total, 1686 (65.1%) of the DEGs were ‘Human UP’ and 901 (34.9%) of the DEGs were ‘Chimp UP’ genes (Fig. 2B; Table S1).

Hippocampal neurodevelopmental markers *PAX6*, *OTX2*, *NEUROD1* and *FOXG1* were found to be highly expressed in both species (Figs 1B,C, 2B).

The ‘Human UP’ genes include *FOXP2*, *MTRNR2L8*, *DHX40*, *VPS13B*, *WDFY2* and *PURB*, all of which are associated with neurodevelopment or neurodegenerative disease (Abrajano et al., 2009; Hickey et al., 2019; Kamboh et al., 2019; Kolehmainen et al., 2003; MacDermot et al., 2005; Mathys et al., 2019; Sin et al., 2015; Taher et al., 2014). The ‘Chimp UP’ genes include *HIST1H3A*, *BCL2L2* and *CLIC1*, which have been reported to be expressed in the hippocampus and are associated with sleep, AD and neurite outgrowth (Averaimo et al., 2014; Datson et al., 2009; Wei, 2020).

To understand the transcriptional programs driving these differences in gene expression, we conducted an Ingenuity pathway analysis. Three of the top five upstream regulators predicted by the pathway analysis were the transcription factors CREB1, FOXA2 and TBR2 (Fig. 2C). CREB1 is known to regulate genes involved in the nervous system and neurodevelopment (reviewed by Sakamoto et al., 2011), FOXA2 controls dopaminergic neuronal development and disease (Kittappa et al., 2007), and TBR2 plays a crucial role in cortical and hippocampal neurogenesis and is the signature marker of the intermediate progenitor cell population (Cipriani et al., 2016; Englund, 2005). This pathway analysis was consistent with the RNA-seq data as predicted targets of all three transcription factors were also found to be among the 2588 genes differentially expressed between human and chimpanzee hpIPCs (Fig. 2D; Tables S2-S4). The pathway analysis also determined that several of the DEGs are involved in embryonic development (Fig. 2D; Table S5). Overall, these findings indicate that previously characterized neurodevelopmental gene regulatory networks are utilized differently during human and chimpanzee hippocampal development.

### Human-specific chromatin accessibility patterns in hpIPCs

After identifying DEGs and the transcriptional networks that may be involved, we sought to identify cis-regulatory differences between the human and chimpanzee hpIPCs. To this end, we conducted ATAC-seq on the hpIPCs from both species. We used the same batches of differentiated iPSCs for the ATAC-seq as we did for the RNA-seq (i.e. from the same batch of differentiation) and generated 100 bp long paired-end Illumina reads.

**Fig. 3.**
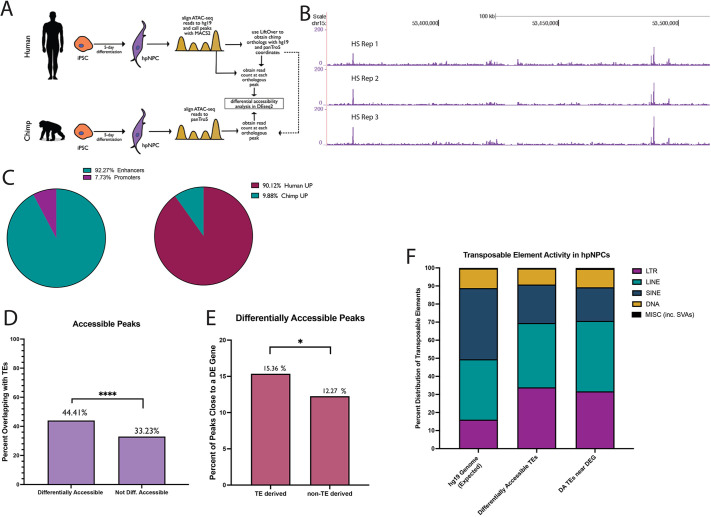
**Human-centric chromatin accessibility analysis.** (A) Schematic of iPSC differentiation followed by ATAC-seq library generation and analysis. (B) UCSC Genome Browser visualization of human ATAC-seq libraries (HS Reps 1, 2 and 3) aligned to hg19 genome assembly. (C) Distribution of the DA peaks into enhancers or promoters, and into ‘Human UP’ (greater accessibility in humans) or ‘Chimp UP’ (greater accessibility in chimpanzees). (D) DA chromatin regions (*P*<0.05, *n*=3006) are more likely to overlap with transposable elements (TEs) than non-DA regions (*P*>0.9, *n*=3006). (E) The 1335 TE-derived DA regions are more likely to be near a DEG, compared with DA regions that do not overlap a TE. *P<0.05; ****P<0.0001 (Fisher's exact test). (F) Distribution of the five major TE classes in the human genome, compared with their distribution among the TE-derived DA chromatin regions, and among the TE-derived DA chromatin regions proximal to a DEG.

We first performed a human-centric analysis. We aligned the ATAC-seq reads from all six cell lines to the respective reference genome assemblies (hg19 for the human cell lines, panTro5 for the chimpanzee cell lines) and only retained uniquely mapped reads with high mapping quality (Samtools *q*=10 filtering). Next, we identified regions of accessible chromatin (peaks; FDR<0.05) in all three human cell lines. Only peaks replicated in all the three human lines were retained. To carry out a proper comparison, we only retained replicated human ATAC-seq peaks with orthologs in the chimpanzee genome (see Materials and Methods; Fig. 3A). This filtering ultimately resulted in 82,235 human ATAC-seq peaks that were replicated in all the human cell lines and with orthologs in the chimpanzee genome. These 82,235 regions were used for downstream analysis. We found that the chromatin accessibility at these regions was highly reproducible across all three human cell lines (Fig. 3B).

Next, we quantified the ATAC-seq read depth for each of the 82,235 regions and used DESeq2 to identify sites exhibiting differential chromatin accessibility between the two species. In total, we identified 3006 DA regions [FDR<0.05; log_2_(fold change)>1.5 or <−1.5; Tables S6 and S7]. Of these regions, 92.3% were located at least 1 kb away from the closest transcription start site (TSS), suggesting that they could be putative enhancers, whereas the remaining were putative promoters (Fig. 3C). As expected, given that this analysis was performed with a human-centric approach, 90.1% of the DA peaks were significantly more accessible in the human hpIPCs relative to chimpanzee hpIPCs (‘Human UP’; Fig. 3C).

As TE insertions can be a source of cis-regulatory evolution, we examined whether the DA regions were more likely to overlap with a TE than those that were accessible to the same degree in both species (non-DA; Fig. 3D; Tables S9 and S10). We observed that 1335/3006 (44.4%) DA regions overlapped a TE (Table S8). This is significantly higher than what was observed for the non-DA peaks (33.2% overlapped a TE; two-sided Fisher's exact test, *P*<0.0001; Fig. 3D). This indicates that chromatin regions with human-specific accessibility are significantly more likely to be TE-derived than the regions with accessibility levels conserved between human and chimpanzee.

Next, we associated the nearest gene to each DA region and found that TE-derived DA regions were significantly more likely to be near a DEG relative to non-TE derived DA regions (two-sided Fisher's exact test, *P*=0.016; Fig. 3E). Finally, we investigated whether specific TE families were overrepresented among the TE-derived DA regions and found enrichment for LTRs. Although LTRs account for ∼16% of all human annotated TEs, they represented 33.9% of the TEs overlapping DA regions in our human-centric analysis (two-Sided Fisher's exact test, *P*<0.0001; Fig. 3F; Table S11). Of these enriched LTRs, 97.1% were ERVs. Notably, 31.7% of the LTR-derived DA regions were located near a DEG (two-sided Fisher's exact test *P*<0.0001).

Taken together, these data indicate that there are TE-derived cis-regulatory elements that have significantly greater accessibility in humans than in chimpanzees during hippocampal neurogenesis. These TE insertions preceded the human-chimpanzee split, but the difference in accessibility is species specific, suggesting that the co-option into gene regulatory networks took place after the human-chimpanzee divergence.

### Chimpanzee-specific chromatin accessibility patterns in hpIPCs

**Fig. 4.**
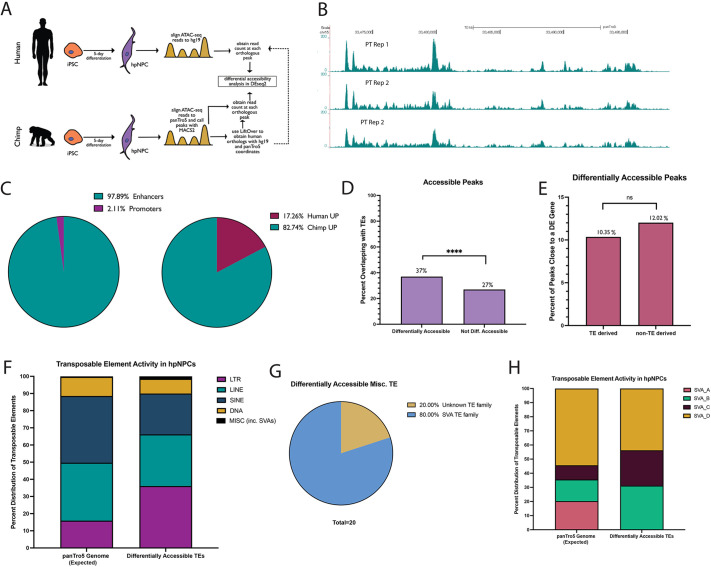
**Chimpanzee-centric chromatin accessibility analysis.** (A) Schematic of iPSC differentiation followed by ATAC-seq library generation and analysis. (B) UCSC Genome Browser visualization of human ATAC-seq libraries (PT Reps 1, 2 and 3) aligned to hg19 genome assembly. (C) Distribution of the DA peaks into enhancers or promoters, and into ‘Human UP’ (greater accessibility in humans) or ‘Chimp UP’ (greater accessibility in chimpanzees). (D) DA chromatin regions (*P*<0.05, *n*=3806) are more likely to overlap with transposable elements than other accessible regions (*P*>0.9, *n*=3806). (E) Distribution of DA regions that overlap a TE and proximity to genes differentially expressed between human and chimpanzee hpIPCs. ns, not significant; ****P<0.0001 (Fisher's exact test). (F) Distribution of the five major TE classes in the chimpanzee genome, compared with their distribution among the 1410 TE-derived DA chromatin regions. (G) Breakdown of the 20 miscellaneous TEs represented in the 1410 TE-derived DA chromatin regions. (H) Distribution of SVA family of TEs in the human genome compared with their distribution in the TE-derived DA chromatin regions (*n*=16).

We repeated the ATAC-seq analysis as described above, but this time with a chimpanzee-centric approach. We started from a set of 72,211 peaks found to be replicated in all the three chimpanzee lines and with orthologs in both species (Fig. 4A,B). With this approach, we identified 3806 ATAC-seq peaks as being DA between human and chimpanzee [FDR<0.05; log_2_(fold change)>1.5 or <−1.5; Tables S16 and S17], 82% of which displayed greater accessibility in chimpanzee compared with humans (i.e. ‘Chimp UP’; Fig. 4C). Similar to what we observed with the human-centric analysis, 97.9% of the 3806 DA regions were putative enhancers (distance>1 kb from TSS; Fig. 4C).

As seen in the human-centric analysis, chimpanzee-specific DA peaks were more likely to be TE derived than those that were similarly accessible across species (two-sided Fisher's exact test, *P*<0.0001; Fig. 4D; Tables S18-S20). Of the chimpanzee DA regions, 37.1% overlapped an annotated chimpanzee TE, compared with only 28.1% of the non-DA regions (two-sided Fisher's exact test, *P*<0.0001; Fig. 4D). However, the TE-derived enhancers in this chimpanzee-centric analysis were no more likely to be located near DEGs than the non-TE derived ones (Fig. 4E).

We found enrichment for LTRs, which account for approximately 16% of the chimpanzee TEs but represented 36.1% of the TE-derived DA regions (two-sided Fisher's exact test, *P*-value<0.0001; 98.2% were ERVs; Fig. 4F; Table S21), and SVAs, which account for just 0.25% of annotated chimpanzee TEs but represented 1.1% of the TE-derived DA regions (two-sided Fisher's exact test, *P*<0.0001, Fig. 4G; Table S23). In particular, the SVA-B and SVA-C subfamilies were the most enriched (Fig. 4H). Taken together, these data indicate that chimpanzee ERV and SVA transposons were co-opted into regulatory elements important for the developing chimpanzee hpIPCs.

### Genomic features underlying species-specific LTR enrichment at hpIPC enhancers

**Fig. 5.**
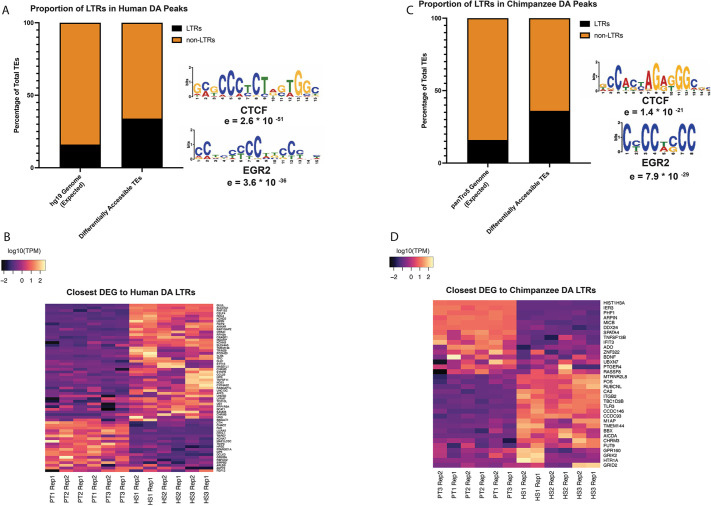
**LTRs are enriched among human and chimpanzee DA transposons.** (A) Distribution of LTRs compared with non-LTRs in the human genome and in the 1335 human TE-derived DA regions, with predicted binding motifs. (B) Differentially expressed genes close to the human-enriched DA LTRs. (C) Distribution of LTRs compared with non-LTRs in the chimpanzee genome and in the 1410 chimpanzee TE-derived DA regions, with predicted binding motifs. (D) Differentially expressed genes close to the chimpanzee-enriched DA LTRs.

We aimed to further investigate genomic features potentially underlying the LTR enrichment among the DA hippocampal progenitor regions. To this end, we conducted a motif analysis using the MEME suite (Bailey et al., 2015). Binding motifs for CTCF and EGR2 were detected as being enriched in the LTR-derived DA regions identified from both the human-centric and chimpanzee-centric analyses (Fig. 5A,C). *EGR2* is an early response gene involved in learning and memory, in the brain response to stimuli and in hippocampal synaptic plasticity (Cheval et al., 2012; Mukherjee et al., 2021; Poirier et al., 2007). CTCF, a well-known regulator of chromatin structure, has been implicated in various neurodevelopmental disorders (reviewed by Davis et al., 2018).

We identified 63 DEGs located near the human-enriched LTRs (Fig. 5B; Table S12). These included *GLUL*, a glutamine synthetase hypothesized to provide neuroprotection in AD patients, (Kohane and Wood, 2021 preprint) and *DLK1*, a Notch ligand involved in sub-ventricular zone neurogenesis (Ferrón et al., 2011), both of which are upregulated in human hpIPCs compared with chimpanzee hpIPCs.

We identified 34 DEGs located near the chimp-enriched LTRs (Fig. 5D; Table S22). For the chimp-centric analysis, the DEGs located near TE-derived enhancers with species-specific accessibility included *HIST1H3A* and *MTRNR2L8*, which were highly upregulated in the chimpanzee and human hpIPCs, respectively (Fig. 5D). Importantly, *HIST1H3A* has been associated with autism spectrum disorders and sleep deprivation (Crawley et al., 2016; Wei, 2020), whereas *MTRN2L8* has been reported as being upregulated in AD patients (Mathys et al., 2019).

### Human-specific SVAs play a major role in hippocampal neurogenesis

**Fig. 6.**
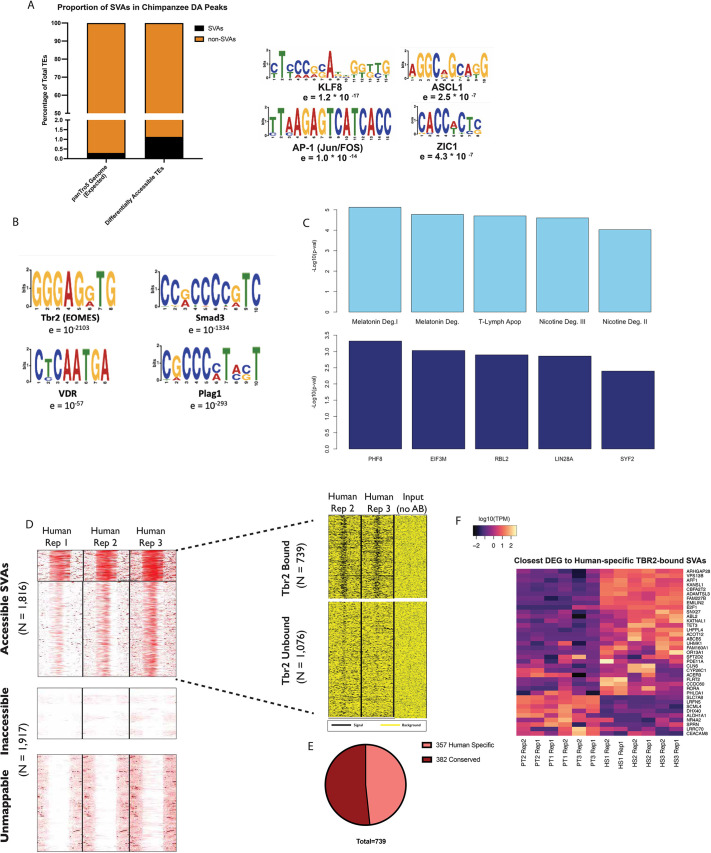
**Human-specific SVAs bind TBR2 and influence neurodevelopment.** (A) The SVAs enriched within the chimpanzee DA peaks are enriched for binding motifs of neurodevelopmental transcription factors KLF8, ZIC1 and ASCL1, as well as the AP-1 dimer. (B) Human SVAs are enriched for the binding motif of TBR2. (C) Genes proximal to human-specific SVAs are predicted to be regulated by neurodevelopmental transcription factors and function in important neuronal pathways. (D) In humans, 1816 SVAs exhibit ATAC-seq signal in hpIPCs (red, signal; white, noise) and, of these, 739 exhibit a TBR2 ChIP signal (black, signal; yellow, background). (E) Breakdown of 739 accessible, TBR2-bound SVAs that are human specific or conserved in chimpanzees. (F) Differentially expressed genes close to the human-specific TBR2-bound SVAs.

As mentioned earlier, the chimpanzee-centric analysis also identified SVA transposons as being enriched within chimpanzee-specific enhancers (Fig. 6A; Table S23). These SVAs were enriched for the identified binding motifs of the neurodevelopmental factors ASCL1, ZIC1 and KLF8 (Andersen et al., 2014; Aruga, 2004; Yi et al., 2014), as well as the JUN/FOS-AP-1 dimer, which is a known enhancer activator (Raivich, 2008; Raivich and Behrens, 2006) (Fig. 6A).

It is important to note that all the analyses shown so far were exclusively based on genomic sites with characterized orthologs in both species, to ensure an equivalent comparison. However, nearly 2000 SVA copies, including the entire SVA-E and SVA-F subfamilies, are exclusive to the human genome. Given that previous studies found that SVAs are highly enriched in active enhancers and promoters of the human hippocampus (Trizzino et al., 2018), we sought to investigate this further. We focused on the SVA copies exclusively present in the human genome and not in any other primate genome (hereafter human-specific SVAs). We performed sequence-based motif analysis for all the human-specific SVAs and identified the binding motif for the hpIPC signature factor TBR2 as the most enriched (*P*=10^−2103^; Fig. 6B). Motifs for other transcription factors associated with hippocampal neurogenesis and function were also recovered (SMAD3, VDR and PLAG1; Fig. 6B).

Next, we annotated all genes found within 50 kb from each human-specific SVA. A total of 2216 genes were recovered using this approach. We performed pathway analysis on this set of genes and found that ‘melatonin degradation’ and ‘nicotine degradation’ were the two most significantly enriched pathways (*P*=7.6×10^−6^ and *P*=2.5×10^−5^, respectively; Fig. 6C) and PHF8 was recovered as the top upstream regulator for the gene network (Fig. 6C). Notably, both melatonin and nicotine degradation pathways are strongly active in the human hippocampus. PHF8 is a histone demethylase that contributes to the regulation of mTOR. The mTOR pathway is hyperactive in the human hippocampus where it regulates the protein synthesis-dependent plastic changes underlying learning and memory (Bekinschtein et al., 2007; Fortress et al., 2013; Graber et al., 2013). Mutations in the *PHF8* gene cause cognitive impairment and intellectual disability (Chen et al., 2018).

Therefore, we sought to determine the contribution of human-specific SVAs to the TBR2-mediated gene regulatory network in human hpIPCs. Using our ATAC-seq data, we identified 1816 human SVAs as accessible in human hpIPCs (Fig. 6D). Of these, nearly a quarter (434) displayed high accessibility, whereas 1382 were moderately accessible (Fig. 6D). Next, we used ChIP-seq to profile TBR2 binding in two human lines at day 5 of hpIPC differentiation. As with the previous sequencing experiments, we generated 100 bp long paired-end reads and only retained uniquely mapping high-quality reads (Samtools *q*=10 filtering) in order to maximize the chance to properly map reads on repetitive regions. This experiment revealed that 739 of the accessible SVAs showed TBR2 signals in the two human lines (Fig. 6D; Table S13). Notably, 257 (48.3%) of the TBR2-bound SVAs were human specific. (Fig. 6E; Table S14). TBR2-bound human-specific SVAs were located near 37 genes that our RNA-seq analysis identified as being differentially expressed between human and chimpanzee (Fig. 6F; Table S15). These genes include *VPS13B* (upregulated in humans), which is responsible for a rare developmental disease known as Cohen syndrome (Kolehmainen et al., 2003); *NR4A2* (downregulated in humans), which has been implicated in neurodevelopmental language impairment (Reuter et al., 2017); *DHX40* (downregulated in humans), which is implicated in AD (Taher et al., 2014); and *E2F1* (upregulated in humans), which is a cell-cycle regulator associated with several neurodegenerative diseases such as AD (Zhang et al., 2010).

In summary, these data support a model in which human-specific SVAs provided a substrate for binding sites of TBR2 and other important hippocampal regulators. Therefore, it is likely that they were co-opted in the gene regulatory networks that are active during hippocampal neurogenesis, which led to human-specific regulation of key genes.

### CRISPR-mediated SVA repression has massive repercussions on global gene expression

**Fig. 7.**
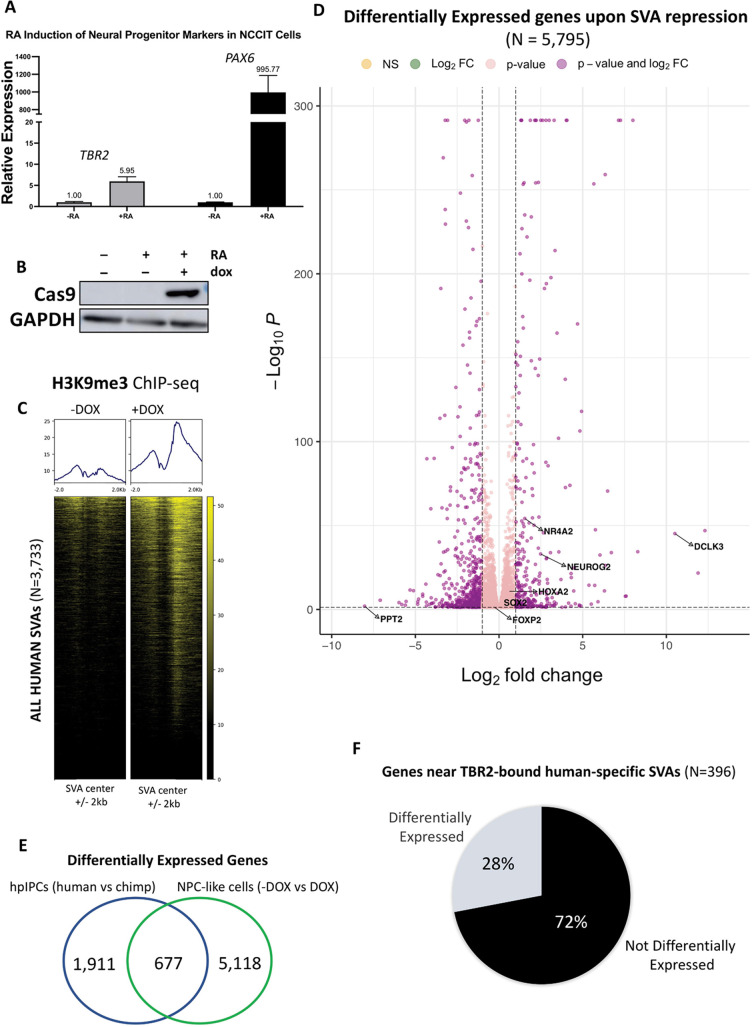
**CRISPR interference of human SVAs.** (A) Treating NCCITs with RA for 7 days led to the induction of *TBR2* and *PAX6* expression, as shown by RT-qPCR. (B) Treating the RA-NCCIT stable CRISPR line for 72 h with doxycycline (dox) led to dCas9 activation, as shown in the Cas9 immunoblot. Images are representative of two replicates. (C) dCas9 activation resulted in the deposition of H3K9me3 at most human SVAs as shown in the H3K9me3 ChIP-seq heatmap. (D) Volcano plot depicting genes differentially expressed upon doxycycline treatment. (E) Venn diagram showing overlap between genes that are differentially expressed between human and chimpanzee hpIPCs and between RA-NCCITs with or without doxycycline treatment (i.e. with and without SVA repression). (F) Pie chart illustrating the fraction of TBR2-controlled genes that are differentially expressed upon SVA repression in RA-NCCITs.

To further assess the contribution of SVA transposons to the gene regulation of hpIPCs, we leveraged CRISPR interference to simultaneously repress most of the active SVAs. We used NCCIT cells treated with retinoic acid (RA) as the experimental system for this purpose. The NCCIT cell line is derived from embryonal carcinoma and thus exhibits a gene expression signature highly similar to human embryonic stem cells (Fuentes et al., 2018; Barnada et al., 2022). Importantly, NCCITs treated for 7 days with RA differentiate into intermediate neural progenitor-like cells expressing both PAX6 and TBR2 (Mandal et al., 2015; Fig. 7A). RNA-seq data [transcripts per million (TPMs)] suggest very strong correlation between RA-treated NCCITs and human day-5 hpIPCs (Pearson correlation *P*<2.2×10^−16^, R=0.99). We cloned a stable NCCIT line with a permanently incorporated doxycycline-inducible, catalytically dead Cas9 fused to a repressive KRAB domain (dCas9-KRAB). The KRAB domain deposits repressive histone methylation (H3K9me3) to the regions targeted by dCas9 via single guide RNAs (sgRNAs). Into this same line, we also permanently knocked in two sgRNAs that are able to target >80% of all SVAs (Pontis et al., 2019). Hereafter, we refer to the RA-treated CRISPR line as RA-NCCITs. Remarkably, exposing the RA-NCCITs to doxycycline for 72 h was sufficient to induce dCas9 activation (Fig. 7B) and the deposition of the repressive H3K9me3 on over 2500 previously unmethylated SVAs (Fig. 7C). We next performed RNA-seq on the RA-NCCITs with or without doxycycline treatment (three replicates per condition). First, we observed that the genome-wide expression levels (TPMs) of the RA-NCCITs were highly correlated with those of the iPSC-derived human hpIPCs (Pearson correlation=0.93; *P*<2.2×10^−16^). This indicates that RA-treated NCCITs are appropriate to model hpIPCs.

Then, we compared the expression levels of the RA-NCCITs with or without doxycycline treatment and identified 5795 DEGs (FDR<0.05; Fig. 7D; Table S24). Of these genes, 677 were previously identified as differentially expressed upon comparing human with chimpanzee hpIPCs (Fig. 7E; Table S25). In other words, the expression of over a quarter (26.1%) of the genes that exhibited a human-specific expression signature in hpIPCs seem to be under the control of SVA transposons. Importantly, one of these genes is *FOXP2*, which has been associated with the evolution of language and is implicated in several speech disorders (Enard, 2011; Liégeois et al., 2016; MacDermot et al., 2005).

As the guide RNAs used for this experiment were originally designed to target a DNA sequence shared by the SVAs with the LTR5H family (Pontis et al., 2019), we restricted the analysis to the genes that are associated with human SVAs (i.e. only considering the genes that represent the closest gene to an annotated SVA; hereafter SVA genes). By doing so, we found 611 SVA genes as differentially expressed in RA-NCCITs upon SVA repression. Of these, 90 were previously identified as differentially expressed upon comparing human with chimpanzee hpIPCs. Thus, the expression of these 90 genes can be bona fide considered to be directly regulated by SVA-derived enhancers in both primary hippocampal progenitors and in the NCCIT cell line. Remarkably, the large majority of these genes (72.5%) had decreased expression upon SVA repression. These include *SOX2*, *FGF2*, *PRDM1*, *NTRK2* and *TFAP2B*.

Finally, we examined the genes previously identified as being near a TBR2-bound human-specific SVA in iPSC-derived hpIPCs and found that nearly a third of them lose expression in RA-NCCITs upon SVA repression (Fig. 7F). In summary, our functional experiments indicate a widespread role for human-specific SVA transposons as cis-regulatory elements during hippocampal neurogenesis.

## DISCUSSION

The hippocampus is susceptible to specific neurodegenerative disorders such as AD but may have also played an important role in the evolution of human cognition. Spatial memory, which is attributed to the hippocampus, may have contributed to the geographic expansion of ancient humans. Characterizing the human-specific gene regulatory networks of hippocampal development provides insight into its role in human evolution. Although there is no consensus on whether the cognitive phenotypes seen in AD are uniquely specific to humans, understanding the unique properties of the human hippocampus may lead the way to potential treatments. Thus, the work described here is relevant both in terms of evolutionary developmental biology and evolutionary medicine.

To study the evolution of the human hippocampus from a developmental standpoint, we investigated the extent to which TEs contributed to gene expression profiles of human and chimpanzee hpIPCs. TEs account for nearly 50% of the human genome and many elegant studies have established that at least a fraction of TEs can regulate host genes in humans and other primates (Chuong et al., 2013, 2016; Cosby et al., 2021; del Rosario et al., 2014; Du et al., 2016; Fuentes et al., 2018; Jacques et al., 2013; Judd et al., 2021; Lynch et al., 2011, 2015; Mika et al., 2021; Modzelewski et al., 2021; Okhovat et al., 2020; Rayan et al., 2016; Schmidt et al., 2012; Sundaram et al., 2014; Trizzino et al., 2017, 2018; Ward et al., 2018; Xie et al., 2013).

HpIPCs are a transient progenitor population during a crucial developmental stage in the sub-ventricular zone of both the hippocampus and neocortex (Bulfone et al., 1999; Cipriani et al., 2016; Englund, 2005; Kimura et al., 1999), and there is a consensus that this progenitor population may have played a role in the evolution of brain volume in mammals (Florio and Huttner, 2014; Martínez-Cerdeño et al., 2006). To study the developmental evolution of human hpIPCs, we leveraged a comparative approach centered on differentiating human and chimpanzee iPSCs into a neuronal population that closely recapitulates differentiation into hpIPCs, as demonstrated by the high expression of signature markers such as TBR2 and PAX6.

The transcriptomes of human and chimpanzee hpIPCs have not been previously compared, largely due to the limited availability of primary tissue. Here, we carried out this comparison using our iPSC-derived system and identified profound differences between the two species, with over 2500 genes differentially expressed at this stage. These genes include several that were previously associated with cognitive function, language, neurodevelopment and neurodegeneration, many of which are upregulated in humans relative to the chimpanzee (e.g. *MTRNR2L8*, *DHX40*, *VPS13B*, *WDFY2* and *PURB*).

We demonstrate that species-specific enhancers significantly contributed to the gene expression differences that we identified. This is consistent with recent studies that used primary brain tissues from several species to profile species-specific cis-regulatory activity in mammals (Emera et al., 2016; Reilly et al., 2015). Importantly, we found that these species-specific enhancers are enriched for young transposable elements. Several studies have identified evolutionarily young L1 long interspersed nuclear elements (LINEs) as active in the brain during different developmental stages, suggesting that they could serve as alternative promoters for many genes involved in neuronal functions (Coufal et al., 2009; Jönsson et al., 2019; Sur et al., 2017; Thomas et al., 2012; Zhao et al., 2019). Here, we identified other young transposable elements, ERVs and SVAs, as regulators of IPC gene expression. The identification of ERVs as candidate enhancers in human and chimpanzee IPCs is consistent with previous studies that demonstrated that ERVs heavily impact gene regulatory programs during the immune response (Chuong et al., 2013, 2016), in pluripotency maintenance and development (Coluccio et al., 2018; Fuentes et al., 2018; Miao et al., 2020), in the mammalian placenta (Lynch et al., 2011, 2015; Mika et al., 2021), in the primate liver (Trizzino et al., 2017) and in many cancer types (Ito et al., 2020; Ivancevic and Chuong, 2020; Shah et al., 2021). Similar to the L1s, the ERVs also possess a well-defined cis-regulatory architecture (e.g. they have their own promoter), and this may have played a role in the co-option of these TEs as functioning cis-regulatory elements.

The SVAs are particularly interesting from a human evolution standpoint, given that half the known copies are exclusively present in our species. Moreover, SVAs are among the few transposable elements that still exhibit active transposition in the human genome. We and others have previously revealed that SVAs can be sources of enhancers in primates (Playfoot et al., 2021; Pontis et al., 2019; Trizzino et al., 2017, 2018). The repression of some SVAs by specific zinc-finger proteins at specific stages of neuronal development is also a crucial mechanism for successful neurogenesis (Playfoot et al., 2021; Pontis et al., 2019; Turelli et al., 2020). Here, we demonstrate that SVAs are pervasive regulators of hippocampal neurogenesis and they act as enhancers in the hpIPC population. By using CRISPR interference, we show that repressing hundreds of normally ‘de-repressed’ SVAs alters the expression of thousands of genes. Intriguingly, our CRISPR interference experiments revealed that global SVA repression leads to the attenuation of the expression levels of over a quarter of the ∼2500 genes previously identified as showing human-specific expression in hpIPCs. These include crucial neurodevelopmental regulators such as *FOXP2*, *HAND2*, *MEF2C*, *SOX2* and *SOX4*. In normal conditions, these genes are more highly expressed in humans relative to in chimpanzees, but upon SVA repression, these differences were diminished.

In conclusion, our findings indicate that the development of hippocampal neurons has been profoundly affected by the domestication of young transposable elements. These young TEs have been co-opted as functional enhancers and promoters and ultimately rewired the expression of hundreds of crucially important neuroregulators in the developing human hippocampus. The human-specific gene expression and the associated TE-derived enhancers that we identified here may play important roles in both human evolution and neurodegenerative disease.

## MATERIALS AND METHODS

### Antibodies

The following antibodies were used in this study: anti-TBR2 (15 µg per ChIP, Abcam, ab216870), anti-H3K9me3 (3 µg per ChIP, Abcam, ab8898), anti-H3K21ac (3 µg per ChIP, Abcam, ab4729), anti-TBR2 (1:500 for western blotting, Santa Cruz Biotechnology, sc-293481), anti-GAPDH (1:1000 for western blotting, Cell Signaling Technology, 5174), HRP-conjugated anti-rabbit (1:10,000 for western blotting, Cell Signaling Technology, 7074S) and HRP-conjugated anti-mouse (1:10,000 for western blotting, Cell Signaling Technology, 7076S).

### Human and chimpanzee iPSC cultures

The human male iPSC line denoted as SV20 was obtained from the University of Pennsylvania, where it was generated, and validated by the expression of pluripotency markers and differentiation into various cell types in multiple studies (Pagliaroli et al., 2021; Pashos et al., 2017; Yang et al., 2015; Zhang et al., 2015). The human female iPSC line GM 23716 was obtained from the Coriell Institute for Medical Research (Camden, NJ, USA) and validated by the expression of pluripotency markers and differentiation into cranial neural crest cells in a previous study (Pagliaroli et al., 2021). The human female iPSC line 21792 and all three chimpanzee iPSC lines were obtained from the laboratory of Yoav Gilad at the University of Chicago and validated in previous studies (Gallego Romero et al., 2015; Ward et al., 2018).

The iPSC lines were expanded in feeder-free, serum-free mTeSR1 medium (STEMCELL Technologies). Cells were passaged ∼1:10 at 80% confluency using ReLeSR (STEMCELL Technologies) and small cell clusters (50-200 cells) were subsequently plated on tissue culture dishes coated overnight with Geltrex LDEV-Free hESC-qualified reduced growth factor basement membrane matrix (Thermo Fisher Scientific).

### hpIPC differentiation

The iPSC lines were differentiated into hpIPCs as previously described (Yu et al., 2014). Three batches consisting of one human and one chimpanzee iPSC line each were cultured until ∼50-70% confluence was reached, and then treated with the hpIPC medium for 5 days prior to collection for RNA-seq, ATAC-seq, ChIP-seq or immunofluorescence. The hpIPC medium consisted of Dulbecco's modified Eagle medium (DMEM)/F12 (Invitrogen), 0.5× N2 (Invitrogen), 0.5× B27 (Invitrogen), DKK1 (0.5 μg/ml, BioLegend), cyclopamine (1 μM, LC Labroatories), Noggin (0.5 μg/ml, BioLegend), SB431542 (10 μM, Selleck Chemicals) and the antibiotics penicillin/streptomycin (1× from 100× stock, Gibco).

### Western blotting

For western blotting of total lysates, cells were harvested and washed three times in 1× PBS and lysed in RIPA buffer (50 mM Tris-HCl pH 7.5, 150 mM NaCl, 1% Igepal, 0.5% sodium deoxycholate, 0.1% SDS, 500 µM dithiothreitol) with protease inhibitors (Sigma-Aldrich). Approximately 20 μg of whole cell lysates were loaded in Novex WedgeWell 4-20% Tris-Glycine Gels (Invitrogen) in a Tris-Glycine-SDS buffer (Invitrogen) and separated by gel electrophoresis (SDS-PAGE). The proteins were then transferred to Immun-Blot PVDF membranes (Thermo Fisher Scientific) for antibody probing. Membranes were incubated with 10% bovine serum albumin in TBS with 3% Tween 20 (TBST) for 30 min at room temperature (RT), then incubated for variable times with suitable antibodies diluted in 5% bovine serum albumin in 1× TBST, washed with TBST and incubated with a dilution of 1:10,000 of the secondary antibody for 1 h at RT. The antibody was visualized using Super Signal West Dura Extended Duration Substrate (Thermo Fisher Scientific) and imaged with Amersham Imager 680.

### Real-time quantitative PCR

Cells were lysed in TRI-reagent (Invitrogen) and RNA was extracted using the Direct-zol RNA MiniPrep kit (Zymo Research). Approximately 600 ng of template RNA was retrotranscribed into cDNA using RevertAid first strand cDNA synthesis kit (Thermo Fisher Scientific) according to the manufacturer’s directions. Approximately 15 ng of cDNA was used for each real-time quantitative PCR (RT-qPCR) reaction with 0.1 μM of each primer, 10 μl of PowerUp SYBR Green Master Mix (Applied Biosystems) in a final volume of 20 μl, using QuantStudio 3 Real-Time PCR System (Applied Biosystems). Thermal cycling parameters were set as following: 3 min at 95°C, followed by 40 cycles of 10 s at 95°C, 20 s at 63°C, followed by 30 s at 72°C. Each sample was run in triplicate. 18S rRNA was used as a normalizer.

### ChIP-seq

Samples from different conditions were processed together to prevent batch effects. Approximately 15 million cells were cross-linked with 1% formaldehyde for 5 min at RT, quenched with 125 mM glycine, harvested and washed twice with 1× PBS. The pellet was resuspended in ChIP lysis buffer (150 mM NaCl, 1% Triton X-100, 0.7% SDS, 500 μM dithiothreitol, 10 mM Tris-HCl, 5 mM EDTA) and chromatin was sheared to an average length of 200-500 bp, using a Covaris S220 Ultrasonicator. The chromatin lysate was diluted with SDS-free ChIP lysis buffer. For ChIP-seq, 10 µg of antibody (3 µg for H3K27ac) was added to 5 µg of sonicated chromatin along with Dynabeads Protein A magnetic beads (Invitrogen) and incubated at 4°C overnight. On day 2, beads were washed twice with each of the following buffers: mixed micelle buffer (150 mM NaCl, 1% Triton X-100, 0.2% SDS, 20 mM Tris-HCl, 5 mM EDTA, 65% sucrose), buffer 500 (500 mM NaCl, 1% Triton X-100, 0.1% sodium deoxycholate, 25 mM HEPES, 10 mM Tris-HCl, 1 mM EDTA) and LiCl/detergent wash buffer (250 mM LiCl, 0.5% sodium deoxycholate, 0.5% NP-40, 10 mM Tris-HCl, 1 mM EDTA). A final wash was performed with 1× Tris-EDTA (TE) buffer. Finally, beads were resuspended in 1× TE buffer containing 1% SDS and incubated at 65°C for 10 min to elute immunocomplexes. Elution was repeated twice, and the samples were further incubated overnight at 65°C to reverse cross-linking, along with the untreated input (5% of the starting material). On day 3, after treatment with 0.5 mg/ml proteinase K for 1 h at 65°C, DNA was purified with Zymo ChIP DNA Clear Concentrator kit and quantified with QUBIT (Invitrogen).

For all ChIP-seq experiments, barcoded libraries were made with NEB ULTRA II DNA Library Prep Kit for Illumina (New England Biolabs) and sequenced on Illumina NextSeq 500, producing 100 bp paired-end reads.

### ChIP-seq analyses

After removing the adapters with TrimGalore! (https://www.bioinformatics.babraham.ac.uk/projects/trim_galore/), the sequences were aligned to the reference hg19, using Burrows–Wheeler Alignment tool (BWA), with the MEM algorithm (http://bio-bwa.sourceforge.net/bwa.shtml). Uniquely mapping aligned reads were filtered based on mapping quality (MAPQ>10) to restrict our analysis to higher quality and likely uniquely mapped reads, and PCR duplicates were removed. We called peaks for each individual using MACS2 (Heinz et al., 2010) (H3K27ac) or Homer (http://homer.ucsd.edu/homer/) at 5% FDR with default parameters.

### RNA-seq

Cells were lysed in TRI-reagent and total RNA was extracted using Quick RNA Miniprep kit (Zymo Research) according to the manufacturer's instructions. RNA was further quantified using the DeNovix DS-11 Spectrophotometer and the RNA integrity was checked on Bioanalyzer 2100 (Agilent). Only samples with an RNA integrity number above 8.0 were used for transcriptome analysis. RNA libraries were prepared using 1 μg of total RNA input using NEBNext Poly(A) mRNA Magnetic Isolation Module, NEBNext Ultra II Directional RNA Library Prep Kit for Illumina and NEBNext Ultra II DNA Library Prep Kit for Illumina according to the manufacturer's instructions (New England Biolabs).

### scRNA-seq

Cells from both species at each time were first incubated with Accutase (STEMCELL Technologies) at 37°C for 7 min. The cells were collected with DMEM/F12 and centrifuged for 5 min at 150 ***g***. The cells were resuspended in 10% fetal bovine serum (Fisher Scientific) in DMEM and strained with a 40 µm cell strainer to create a single-cell suspension. After confirming >90% viability with a Countess III (Invitrogen), the cells were processed with the 10x Genomics Cell Multiplexing Oligo protocol (https://www.10xgenomics.com/support/single-cell-gene-expression/documentation/steps/sample-prep/cell-multiplexing-oligo-labeling-for-single-cell-rna-sequencing-protocols) and 10x Genomics 3′ Cell Plex Kit.

### scRNA-seq analyses

10× Cell Ranger (Zheng et al., 2017) was used to demultiplex and map the scRNA-seq data, with the tools cellranger multi and cellranger mkfastq. Seurat 4 (Hao et al., 2021) was used for individual analysis of scRNA-seq data, as well as integration of human and chimpanzee datasets. The human genes used to classify the cells into the G1/S/G2/M phases of the cell cycle were obtained from https://github.com/hbctraining/scRNA-seq/blob/master/lessons/06_SC_SCT_and_integration.md.

### RNA-seq analyses

After removing the adapters with TrimGalore!, Kallisto (Bray et al., 2016) was used to count reads mapping to each gene. We analyzed differential gene expression levels with DESeq2 (Love et al., 2014), with the following model: design=∼condition, where condition indicates either Human or Chimpanzee.

### ATAC-seq

For ATAC-Seq experiments, 50,000 cells per condition were processed as described in the original ATAC-seq protocol paper (Buenrostro et al., 2013). ATAC-seq data were processed with the same pipeline described for ChIP-seq, with one modification: all mapped reads were offset by +4 bp for the forward-strand and −5 bp for the reverse-strand. Peaks were called using MACS2 (Zhang et al., 2008).

### Generation of the NCCIT-dCas9KRAB-SVAsgRNA stable cell line

This cell line was generated in our recent study (Barnada et al., 2022). Briefly, dCas9-KRAB was cloned into a piggyBac transposon containing ampicillin and puromycin resistance, which was obtained from Raquel Fueyo at Stanford University. The piggyBac dCas9-KRAB doxycycline-inducible plasmid and a piggyBac transposase (Cell Signaling Technology) were transfected into NCCIT cells (American Type Culture Collection) at ∼70% confluency using a 6:1 ratio of Fugene HD (Promega) for 48 h in ATCC-formulated RPMI medium (American Type Culture Collection). Two days post-transfection, the medium was changed and the transfected cells were selected using 1 µg puromycin in 1 ml medium. A piggyBac transposon plasmid containing two sgRNAs (SVAsgRNA1, 5′-CTCCCTAATCTCAAGTACCC-3′, and SVAsgRNA2, 5′-TGTTTCAGAGAGCACGGGGT-3′; Integrated DNA Technologies) targeting ∼80% of all annotated SVAs in humans (Pontis et al., 2019) and a piggyBac transposase were transfected into the NCCIT-dCas9KRAB cells using a 6:1 ratio of Fugene HD for 48 h in ATCC-formulated RPMI medium. Two days post-transfection, the medium was changed and the transfected cells were selected using 400 µg geneticin in 1 ml of medium in addition to 1 µg puromycin in 1 ml medium. The NCCIT-dCas9KRAB-SVAsgRNA cell line was maintained in ATCC-formulated RPMI medium supplemented with 10% tetracycline-free fetal bovine serum (Omega Scientific), 1% L-glutamine, 1 µg/ml puromycin and 400 µg/ml geneticin and incubated at 5% CO_2_, 20% O_2_ at 37°C.

### RA-induced neuronal differentiation and CRISPR interference of NCCIT-dCas9KRAB-SVAsgRNA cells

The NCCIT-dCas9KRAB-SVAsgRNA cells, at ∼20% confluency, were treated with 10 µM RA (Sigma-Aldrich) in 10 ml medium for 1 week to induce neuronal differentiation. At day 4, the medium was refreshed and the cells were additionally treated with 2 µg doxycycline in 1 ml of medium for 3 days. The cells were collected on day 7 of RA treatment (day 3 of doxycycline treatment) for RT-qPCR and genomic experiments. Expression of the doxycycline-inducible dCas9 was verified via western blotting.

### Statistical and genomic analyses

All statistical analyses were performed using R v3.3.1 or Graphpad Prism version 9.2.0 for Mac OS X. BEDTools v2.27.1 (Quinlan and Hall, 2010) was used for genomic analyses. Pathway analysis was performed with Ingenuity Pathway Analysis Suite (QIAGEN, https://www.qiagenbioinformatics.com/products/ingenuity-pathway-analysis). Motif analyses were performed using the MEME suite (Bailey et al., 2015), and specifically with the MEME-ChIP application. FASTA files of the regions of interest were produced using BEDTools v2.27.1. Shuffled input sequences were used as background. *E*-values<0.001 were used as threshold for significance. Orthologous ATAC-seq regions were identified using the University of California Santa Cruz (UCSC) Genome Browser tool LiftOver.

## Supplementary Material

10.1242/develop.200413_sup1 Supplementary information
