# Supplementary material for: Young transposable elements rewired gene regulatory networks in human and chimpanzee hippocampal intermediate progenitors
Source: Development. 2022 Oct 4;149(19):dev200413. doi: 10.1242/dev.200413 (PMC9641669; doi:10.1242/dev.200413)
Supplement: Supplementary information [file develop-149-200413-s1.pdf]

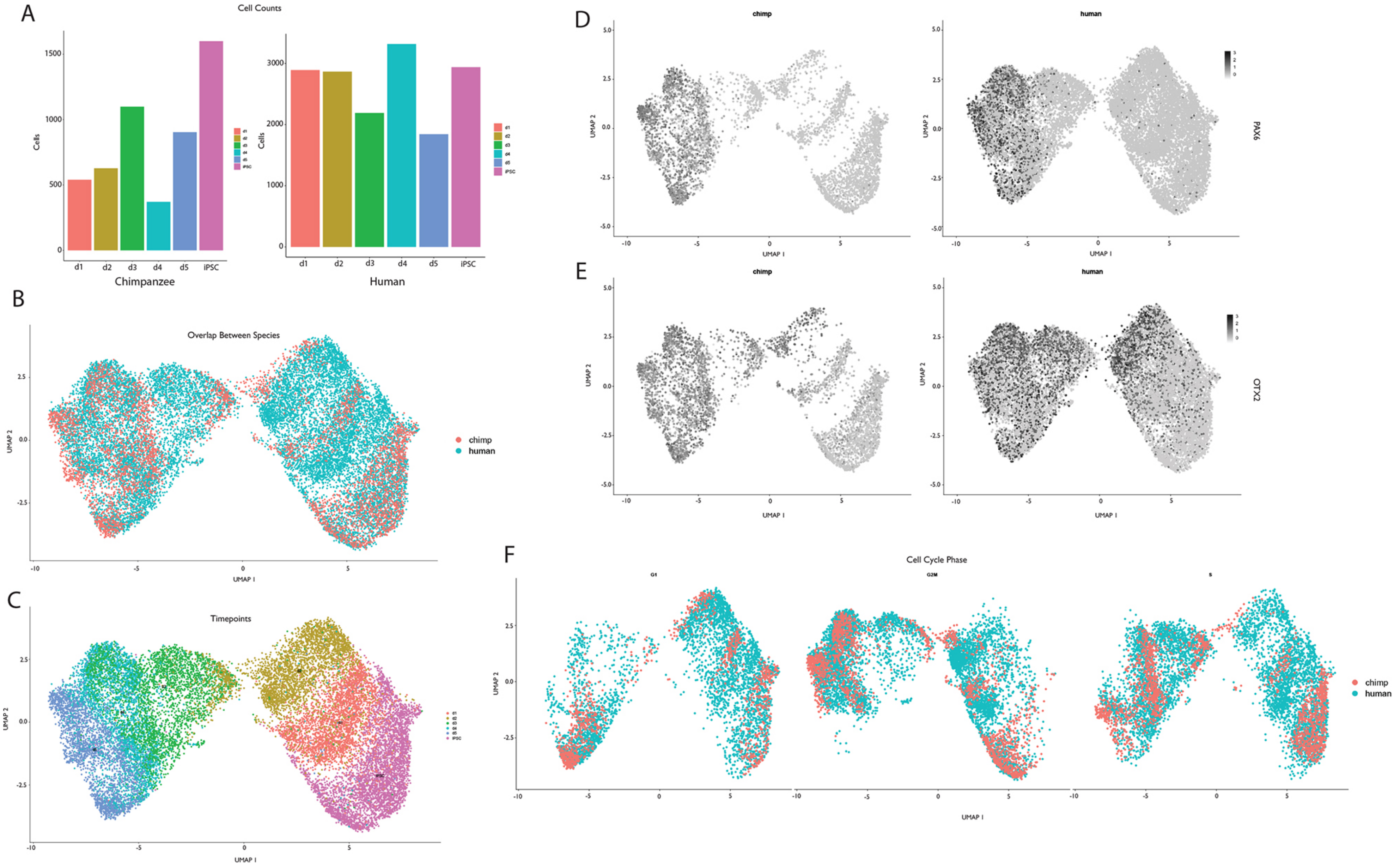

Fig. S1. Single Cell RNA-seq Analysis of Human and Chimpanzee hpIPC Differentiation. (A) Number of cells assayed at each time point in each species (B) Overlap of all human and chimpanzee cells in a single plot. Chimpanze cells are depicted in red and human cells are depicted in blue. (C) Overlap of all human and chimpanzee cells across all six time points in a single plot. (D) Expression of PAX6 across all six time points, human cells are depicted on the right and chimpanzee cells are depicted on the left. (E) Expression of OTX2 across all six time points, human cells are depicted on the left and chimpanzee cells are depicted on the right (F) Overlap of human and chimpanzee cells at G1, G2M and S phases of the cell cycle.

Development • Supplementary information

Table S1. Differentially Expressed Genes
2,588 differentially expressed genes between human and chimpanzee hpIPCs, from DEseq2 analysis with p-value < 0.05 and 2LogFoldChange threshold of 1.5 or -1.5 . P-value and Log2FoldChange is indicated for each gene.

Click here to download Table S1

Table S2. Targets of CREB1
Differentially expressed genes which are observed and predicted targets of CREB1 from Ingenuity Pathway Analysis. P-value and 2Log Fold change is indicated for each gene.

Click here to download Table S2

Table S3. Targets of FOXA2
Differentially expressed genes which are observed and predicted targets of CREB1 from Ingenuity Pathway Analysis. P-value and 2Log Fold change is indicated for each gene.

Click here to download Table S3

Table S4. Targets of TBR2
Differentially expressed genes which are observed and predicted targets of TBR2 from Ingenuity Pathway Analysis. P-value and 2Log Fold change is indicated for each gene.

Click here to download Table S4

Table S5. Embryonic Development Genes
Differentially expressed genes which are observed or predicted to function in the Embryonic Development Network from Ingenuity Pathway Analysis. P-value and 2Log Fold Change is indicated for each gene.

Click here to download Table S5

Table S6. Human Centric Differentially Accessible Chromatin Regions Differentially accessible chromatin regions, from the human centric analysis of the ATAC-seq data.

Click here to download Table S6

Table S7. Coordinates of Human DA Regions

Genomic coordinates of the 3006 DA regions from human centric analysis, from the hg19 genome assembly.

Click here to download Table S7

Table S8. hg19 TE-Derived Peaks

Differentially accessible regions from the human centric ATAC-seq analysis which overlap transposons, along with coordinates for each ATAC-seq region and corresponding transposon sequence.

Click here to download Table S8

Table S9. Control Peaks for Human Centric Analysis

3006 accessible peaks from the human centric analysis with a p-value close to 1, which are not considered differentially accessible and used as statistical controls.

Click here to download Table S9

Table S10. Control Peak Coordinates

Genomic coordinates of the 3006 "non-DA" regions from the human centric analysis

Click here to download Table S10

Table S11. LTR-derived Human Enhancers

Differentially accessible peaks from the human centric analysis which overlap LTR transposons, along with coordinates and the identity of each LTR transposon. This table corresponds to Figure 3 and Figure 5.

Click here to download Table S11

Table S12. Differentially Expressed Genes near Human LTRs

Differentially expressed genes closest to human-enriched LTR transposons, along with the transcript counts normalized to log(TPM+1) for each RNA-seq sample. This table corresponds to Figure 5

Click here to download Table S12

Table S13. TBR2-bound SVA Regions

Coordinates of all TBR2-bound SVA regions identified from ATAC-seq and ChIP-seq. This table corresponds to Figure 6.

Click here to download Table S13

Table S14. Human Specific SVAs

Coordinates of human-specific SVAs bound by TBR2. This table corresponds to Figure 6

Click here to download Table S14

Table S15. Differentially Expressed Genes near Human-specific SVAs

Gene expression data of the genes mentioned in Figure 6

Click here to download Table S15

Table S16. Chimpanzee Centric Differentially Accessible Chromatin Regions Differentially accessible chromatin regions, from the chimpanzee centric analysis of the ATAC-seq data.

Click here to download Table S16

Table S17. Coordinates of Human DA Regions

Genomic coordinates of the 3806 DA regions from chimpanzee centric analysis, from the panTro5 genome assembly.

Click here to download Table S17

Table S18. panTro5 TE-Derived Peaks

Differentially accessible regions from the chimpanzee centric ATAC-seq analysis which overlap transposons, along with coordinates for each ATAC-seq region and corresponding transposon sequence.

Click here to download Table S18

Table S19. Control Peaks for Human Centric Analysis

3806 accessible peaks from the chimpanzee centric analysis with a p-value close to 1, which are not considered differentially accessible and used as statistical controls.

Click here to download Table S19

Table S20. Control Peak Coordinates

Genomic coordinates of the 3806 “non-DA” regions from the chimpanzee centric analysis

Click here to download Table S20

Table S21. LTR-derived Chimpanzee Enhancers

Differentially accessible peaks from the chimpanzee centric analysis which overlap LTR transposons, along with coordinates and the identity of each LTR transposon. This table corresponds to Figure 4 and Figure 5.

Click here to download Table S21

Table S22. Differentially Expressed Genes near Chimpanzee LTRs Differentially expressed genes closest to chimpanzee-enriched LTR transposons, along with the transcript counts normalized to log(TPM+1) for each RNA-seq sample. This table corresponds to Figure 5

Click here to download Table S22

Table S23. Chimpanzee SVAs

Coordinates of the 16 chimpanzee SVAs identified among the chimpanzee DA chromatin regions, in Figure 4.

Click here to download Table S23

Table S24. Differentially Expressed Genes in CRISPRi of SVAs

Genes differentially expressed between control NCCIT-dCas9-KRAB cells (-Doxy) and NCCIT-dCas9-KRAB cells induced with doxycycline (+Doxy), corresponding to Figure 7

Click here to download Table S24

Table S25. hpIPC Genes Controlled by SVAs

Gene expression data for the 677 DEG which overlap between the human vs. chimp and CRISPRi RNA-seq datasets.

Click here to download Table S25
