# Supplementary material for: Young transposable elements rewired gene regulatory networks in human and chimpanzee hippocampal intermediate progenitors
Source: Development. 2022 Oct 4;149(19):dev200413. doi: 10.1242/dev.200413 (PMC9641669; doi:10.1242/dev.200413)
Supplement: Supplementary file 1 [file develop-149-200413_review_history.pdf]

# Young transposable elements rewired gene regulatory networks in human and chimpanzee hippocampal intermediate progenitors

Sruti Patoori, Samantha M. Barnada, Christopher Large, John I. Murray and Marco Trizzino
DOI: 10.1242/dev.200413

**Editor**: Paola Arlotta

**Review timeline**
Original submission: 30 November 2021
Editorial decision: 18 February 2022
First revision received: 15 July 2022
Accepted: 21 August 2022

---

**Original submission**

First decision letter

MS ID#: DEVELOP/2021/200413

MS TITLE: Young transposable elements rewired gene regulatory networks in human and chimpanzee hippocampal intermediate progenitors

AUTHORS: Sruti Patoori, Samantha Barnada, and MARCO TRIZZINO

My apologies for taking this long to get back to you. We received one review very early on but then were never able to collect the additional review that was needed (the reviewers never responded after having accepted to review). This forced us to find additional reviewers, thus the delay. However, I have now received all the referees' reports on the above manuscript, and have reached a decision. The referees' comments are appended below, or you can access them online: please go to BenchPress and click on the 'Manuscripts with Decisions' queue in the Author Area.

As you will see, the referees express considerable interest in your work, but have some significant criticisms and recommend a substantial revision of your manuscript before we can consider publication. If you are able to revise the manuscript along the lines suggested, which may involve further experiments, I will be happy receive a revised version of the manuscript. Your revised paper will be re-reviewed by one or more of the original referees, and acceptance of your manuscript will depend on your addressing satisfactorily the reviewers' major concerns. Please also note that Development will normally permit only one round of major revision.

We are aware that you may be experiencing disruption to the normal running of your lab that make experimental revisions challenging. If it would be helpful, we encourage you to contact us to discuss your revision in greater detail. Please send us a point-by-point response indicating where you are able to address concerns raised (either experimentally or by changes to the text) and where you will not be able to do so within the normal timeframe of a revision. We will then provide further guidance. Please also note that we are happy to extend revision timeframes as necessary.

Please attend to all of the reviewers' comments and ensure that you clearly highlight all changes made in the revised manuscript. Please avoid using 'Tracked changes' in Word files as these are lost in PDF conversion. I should be grateful if you would also provide a point-by-point response detailing how you have dealt with the points raised by the reviewers in the 'Response to Reviewers' box. If you do not agree with any of their criticisms or suggestions please explain clearly why this is so.

© 2022. Published by The Company of Biologists under the terms of the Creative Commons Attribution License (https://creativecommons.org/licenses/by/4.0/).

Reviewer 1

*Advance summary and potential significance to field*

In this manuscript, the authors showed the gene regulatory networks are significantly different between human and chimp hippocampal intermediate progenitors. The authors took advantage of human and chimp iPSC-derived hippocampal progenitors, and analyzed gene expression pattern, chromatin accessibility, and found that transposable elements are act as an enhancer which leads to species-specific gene expression pattern. This study is aiming to address “the evolution of the human hippocampus and the developmental mechanisms driving the human-specific volume increase”, which are indeed unsolved questions and thus important. However, the manuscript did not answer the question, and there are fundamental technical concerns.

*Comments for the author*

Major comments
First of all, it is not clear how do the authors try to explain the mechanisms underlying the size difference of hippocampus among species. Differences in the gene expression pattern are not directly associated with the size difference. One can hypothesize that the differential proliferative capacity of hippocampal intermediate progenitors (hpIPs) in human and chimp leads to the size difference, as the authors implied in the Introduction. However, to the best of my knowledge, there are no studies which shows the proliferative capacity of human hpIP is greater than chimp one. Therefore, it is hard to interpret the present results in the context of hpIP biology and human evolution. The authors need to examine the proliferative capacity of human and chimp hpIPs, and explain the mechanisms underlying the differential proliferative capacity (if this is the case).
Second, it is unclear whether the developmental stages of hpIPs are comparable between human and chimp. Since the timing and speed of neurogenesis is known to be different between human and chimp, the differential gene expression pattern and chromatin accessibility might be explained by the difference in the developmental stage of hpIPs, not by species. The authors need to characterize the IPSC derived hpIPs more, especially the chimp ones. In addition, the protocol the authors utilized can produce both hippocampal granule cells (approx. 60%, doi: 10.1016/j.stemcr.2014.01.009) and pyramidal neurons. If the human and chimp iPSCs produce these two types of hippocampal neurons at different proportion, the bulk RNAseq and ATACseq are not suitable methods. Moreover, the proportion of OTX1 and OTX2-espressing hpNPCs over the total cells was approx. 50% in the previous study (doi: 10.1016/j.stem.2018.04.009). However, the present study (Figure 1B) shows nearly 100% of cells express OTX2. The authors explain why the iPSCs used in the present study differentiate more effectively into the hpNPCs.
Third, the methods regarding the “hpIPC Differentiation” is suboptimally described and it is not enough for reproducing the results. For example, in the results section, the authors described as “we first differentiated one of the human lines for five days”. According to Yu et al., (doi: 10.1016/j.stemcr.2014.01.009), five days post differentiation of iPSCs is still at an embryoid body stage.
Lastly, relating to the second comment, it is not clear whether the authors analyze the hpIPs which produce granule cells or pyramidal cells. Since the developmental process of these two distinct neuronal populations is completely different, and thus the character of their progenitors is different. Therefore, the authors must analyze hpIPs producing granule cells and pyramidal neurons separately.

Minor comments
1. Line 277. Does the differential expression of GLUL indicate the changes in astrocyte differentiation?
2. Line 288. Bath et al., 2012 examined the SVZ for the OB neurogenesis, thus it is misleading.
3. Line 496-497. It is essential to describe the catalog number of the secondary antibodies.
4. Scale bars are essential for Figure 1.
5. It is essential to compare the “genome wide expression levels” among RA-NCCITs, hpIPs and pan NPCs to show RA-NCCITs are indeed a good model for the hpIPs, rather than for pan NPCs. Alternatively, the authors perform dCas9 experiments on the human hpIPs.
6. The abbreviation of “hippocampal intermediate progenitors” should be hpIPs, not hpIPCs, since there is no word for “C”.

© 2022. Published by The Company of Biologists under the terms of the Creative Commons Attribution License (https://creativecommons.org/licenses/by/4.0/).

Reviewer 2

*Advance summary and potential significance to field*

The question of how organ size is controlled on different species is a very relevant one. In the brain in particular there has been profound evolutionary changes in the size of specific brain regions. The current study investigates the mechanisms that potentially led to the expansion of the human hippocampus.
This is a central question in human brain evolution and development.

*Comments for the author*

The authors use stem cell-derived intermediate progenitor cultures to look at a specific possible aspect of hippocampal expansion, the proliferation of progentors. With the assumption that this is the step that affects size (which although likely to play a role is not demonstrated), they make intermediate progenitors from stem cells of apes and humans.

They then look at mechanisms that may control such proliferative differences and highlight the roles of young transposomes as possible regulators of gene expression changes in a specie-specific manner.

The study is interested but it has several limitations, which the authors should address experimentally:
1. Composition of cultures: there is not enough data shown on the cellular composition of these cell cultures. This requires scRNAseq in both apes cultures and human cultures to determine whether the composition of cell types is the same and whether these are cultures that contain ONLY intermediate progenitors.
Any change in either reproducibility of cellular composition or mixed cellular composition in any of these wells could cause the changes in gene expression observed here.
2. Maturity of the cultures: given that different species develop with different speeds, it is possible that the authors are comparing two distinct stages of development in apes vs humans. This needs to be addressed. Comparing trajectories over time rather than single time points may help. All with single cell resolution.
3. Change in gene expression without information on the cell types that may present sch changes is not useful. In other words, scRNAseq is needed to show that indeed one is comparing the same cell types mand maturation states across species.

Overall, this is an interesting MS that askes a very important question but the data are impossible to interpret and reply on unless the points above ar eaddressed.

---

**First revision**

Author response to reviewers' comments

**Reviewer 1 Comments for the Author:**

Major comments

1) First of all, it is not clear how do the authors try to explain the mechanisms underlying the size difference of hippocampus among species. Differences in the gene expression pattern are not directly associated with the size difference. One can hypothesize that the differential proliferative capacity of hippocampal intermediate progenitors (hpIPs) in human and chimp leads to the size difference, as the authors implied in the Introduction. However, to the best of my knowledge, there are no studies which shows the proliferative capacity of human hpIP is greater than chimp one. Therefore, it is hard to interpret the present results in the context of hpIP biology and human evolution. The authors need to examine the proliferative capacity of

© 2022. Published by The Company of Biologists under the terms of the Creative Commons Attribution License (https://creativecommons.org/licenses/by/4.0/).

human and chimp hpIPs, and explain the mechanisms underlying the differential proliferative capacity (if this is the case).

We thank the Reviewer for this comment. First, we would like to emphasize that the goal of the paper is not to explain the mechanisms underlying the size difference of hippocampus among species. Our goal was to compare, genomically, the hippocampal intermediate progenitors between the two species, with particular interest for the role of the transposable elements in the gene regulation of this important progenitor population. Nonetheless, following the Reviewer's suggestion, to investigate any noticeable changes to cell cycle or proliferation between species, we quantified the number of at each phase of the cell cycle within the newly generated single cell RNA-seq data set, based on a set of known cell cycle genes (Figure 1E). Using this, we did not see any differences between species (Figure 1D). Furthermore, we edited the Introduction to better emphasize what the scope of the manuscript is.

2) Second, it is unclear whether the developmental stages of hpIPs are comparable between human and chimp. Since the timing and speed of neurogenesis is known to be different between human and chimp, the differential gene expression pattern and chromatin accessibility might be explained by the difference in the developmental stage of hpIPs, not by species. The authors need to characterize the IPSC derived hpIPs more, especially the chimp ones. In addition, the protocol the authors utilized can produce both hippocampal granule cells (approx. 60%, doi: 10.1016/j.stemcr.2014.01.009) and pyramidal neurons. If the human and chimp iPSCs produce these two types of hippocampal neurons at different proportion, the bulk RNAseq and ATACseq are not suitable methods.

We thank the Reviewer for this consideration, which was shared also by Reviewer-2. To demonstrate that the developmental stages are comparable between the two species, we performed a time-course single-cell RNA-seq experiment, analyzing every differentiation day (iPSC to day-5) in both species. The single-cell RNA-seq experiment demonstrated that the chimp differentiation initially progresses a bit faster along the trajectory than the human data (i.e. chimp day-3 cells seem to align with human day-4), but by day-5, which is the time-point used for all the genomic experiments and analyses, the datasets in the two species align very well (see plot below). Moreover, we conducted a time-course Western Blot in both species and confirmed that TBR2 expression is greatest at days 4 and 5 (Figure 1F), similar to OTX2 and PAX6 expression (Figure 1B, 1C). Lastly, we conducted ChIP-seq against TBR2 and H3K27ac to determine whether at day-5 TBR2 was bound at the same genomic sites in both species (Figure 1G). We identified 3789 TBR2-bound regions in human hpIPCs and observed that many of them also exhibited the active histone mark H3K27ac. Upon translating the coordinates of these regions to the chimpanzee genome, we observed that the 3781 orthologous regions were all bound by TBR2 in chimpanzee hpIPCs. Together, these data indicate that our iPSC-derived model is suitable to study gene regulation within TBR2-positive hippocampal intermediate progenitors in both human and chimpanzee. We hope that these experiments address this important concern The updated figure 1 is reported here for Reviewer's convenience:

© 2022. Published by The Company of Biologists under the terms of the Creative Commons Attribution License (https://creativecommons.org/licenses/by/4.0/).

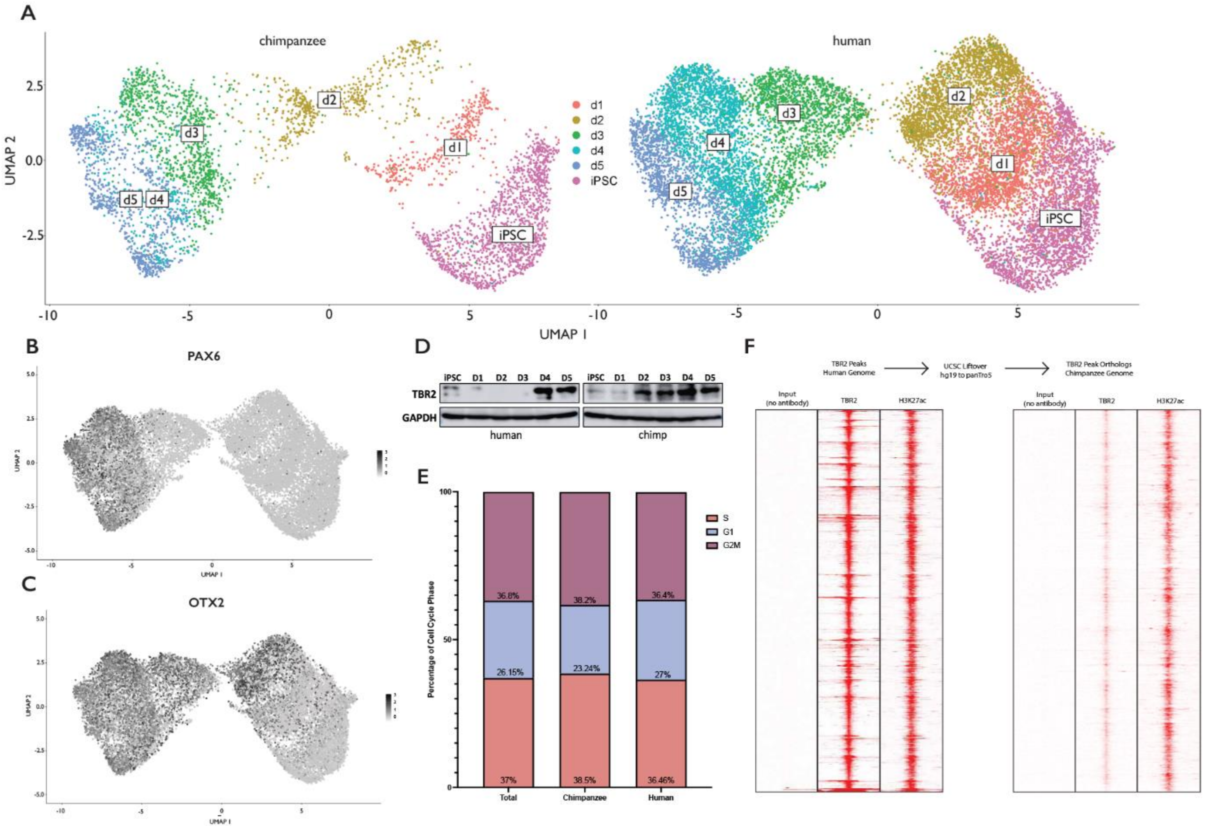


3) Moreover, the proportion of OTX1 and OTX2-espressing hpNPCs over the total cells was approx. 50% in the previous study (doi: 10.1016/j.stem.2018.04.009). However, the present study (Figure 1B) shows nearly 100% of cells express OTX2. The authors explain why the iPSCs used in the present study differentiate more effectively into the hpNPCs.

We leveraged our single-cell RNA-seq data to address this comment. As shown in the new Figure 1C (see above) and in Supplementary Figure 1 (panel E, also reported below for reviewer convenience) the number of cells expressing OTX2 is significantly less than 100% and the levels of expression are variable and heterogeneous across the day-5 cell population.

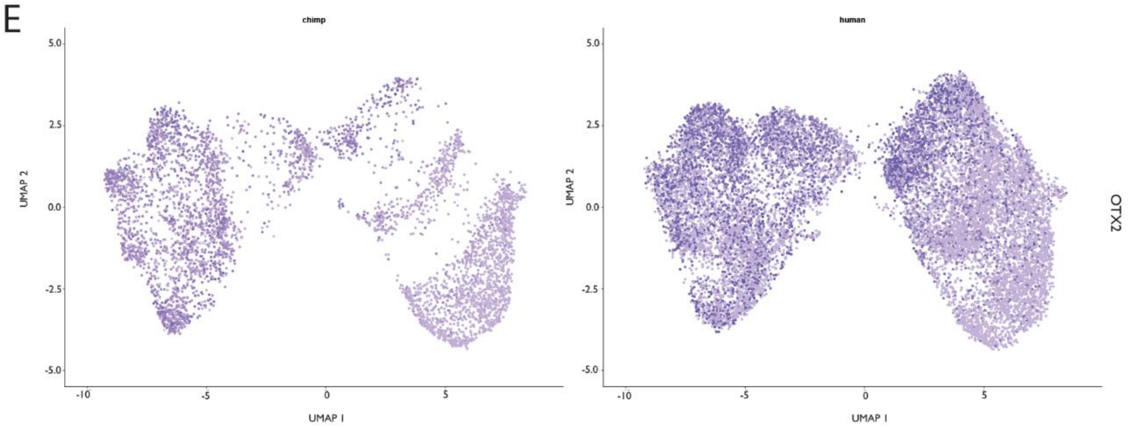


4) Lastly, relating to the second comment, it is not clear whether the authors analyze the hpIPs which produce granule cells or pyramidal cells. Since the developmental process of these two distinct neuronal populations is completely different, and thus the character of their progenitors is different. Therefore, the authors must analyze hpIPs producing granule cells and pyramidal neurons separately.

**We thank the Reviewer for this comment. The same progenitors obtained by Yu et. al 2014 and Sarkar et. al. 2018 can be further induced into DG granule neurons or CA3 pyramidal neurons respectively. The protocol does not give origin to two different progenitor populations.**

© 2022. Published by The Company of Biologists under the terms of the Creative Commons Attribution License (https://creativecommons.org/licenses/by/4.0/).

Minor comments

1. Line 277. Does the differential expression of GLUL indicate the changes in astrocyte differentiation?
   **We did not detect astrocytes in our cell culture.**

2. Line 288. Bath et al., 2012 examined the SVZ for the OB neurogenesis, thus it is misleading.
   **We edited the reference accordingly.**

3. Line 496-497. It is essential to describe the catalog number of the secondary antibodies.
   **Done.**

4. Scale bars are essential for Figure 1.
   **Done.**

5. It is essential to compare the “genome wide expression levels” among RA-NCCITs, hpIPs and pan NPCs to show RA-NCCITs are indeed a good model for the hpIPs, rather than for pan NPCs. Alternatively, the authors perform dCas9 experiments on the human hpIPs.
**Based on balk RNA-seq TPMSs, there is a very high correlation (R=0.99) between RA-NCCITs and hpIPs. We have now mentioned this in the manuscript.**

6. The abbreviation of “hippocampal intermediate progenitors” should be hpIPs, not hpIPCs, since there is no word for “C”.
**The acronym hpIPCs stands for hippocampal Intermediate Progenitor Cells.**

**Reviewer 2 Advance Summary and Potential Significance to Field:**

The question of how organ size is controlled on different species is a very relevant one. In the brain in particular there has been profound evolutionary changes in the size of specific brain regions. The current study investigates the mechanisms that potentially led to the expansion of the human hippocampus. This is a central question in human brain evolution and development.

**Reviewer 2 Comments for the Author:**

The authors use stem cell-derived intermediate progenitor cultures to look at a specific possible aspect of hippocampal expansion, the proliferation of progentors. With the assumption that this is the step that affects size (which although likely to play a role is not demonstrated), they make intermediate progenitors from stem cells of apes and humans. They then look at mechanisms that may control such proliferative differences and highlight the roles of young transposomes as possible regulators of gene expression changes in a specie-specific manner.
The study is interesting but it has several limitations, which the authors should address experimentally:

1. Composition of cultures: there is not enough data shown on the cellular composition of these cell cultures. This requires scRNAseq in both apes cultures and human cultures to determine whether the composition of cell types is the same and whether these are cultures that contain ONLY intermediate progenitors. Any change in either reproducibility of cellular composition or mixed cellular composition in any of these wells could cause the changes in gene expression observed here. 2.Maturity of the cultures: given that different species develop with different speeds, it is possible that the authors are comparing two distinct stages of development in apes vs humans. This needs to be addressed. Comparing trajectories over time rather than single time points may help. All with single cell resolution. 3.Change in gene expression without information on the cell types that may present sch changes is not useful.

In other words, scRNAseq is needed to show that indeed one is comparing the same cell types mand maturation states across species.

**We thank the Reviewer for this consideration, which was shared also by Reviewer-1. To demonstrate that the developmental stages are comparable between the two species, we**

© 2022. Published by The Company of Biologists under the terms of the Creative Commons Attribution License (https://creativecommons.org/licenses/by/4.0/).

performed a time-course single-cell RNA-seq experiment, analyzing every differentiation day (iPSC to day-5) in both species. The single-cell RNA-seq experiment demonstrated that the chimp differentiation initially progresses a bit faster along the trajectory than the human data (i.e. chimp day-3 cells seem to align with human day-4), but by day-5, which is the time-point used for all the genomic experiments and analyses, the datasets in the two species align very well (see plot below). Moreover, we conducted a time-course Western Blot in both species and confirmed that TBR2 expression is greatest at days 4 and 5 (Figure 1F), similar to OTX2 and PAX6 expression (Figure 1B, 1C). Lastly, we conducted ChIP-seq against TBR2 and H3K27ac to determine whether at day-5 TBR2 was bound at the same genomic sites in both species (Figure 1G). We identified 3789 TBR2-bound regions in human hpIPCs and observed that many of them also exhibited the active histone mark H3K27ac. Upon translating the coordinates of these regions to the chimpanzee genome, we observed that the 3781 orthologous regions were all bound by TBR2 in chimpanzee hpIPCs. Together, these data indicate that our iPSC-derived model is suitable to study gene regulation within TBR2-positive hippocampal intermediate progenitors in both human and chimpanzee.

We hope that these experiments address this important concern. The updated figure 1 is reported here for Reviewer’s convenience:

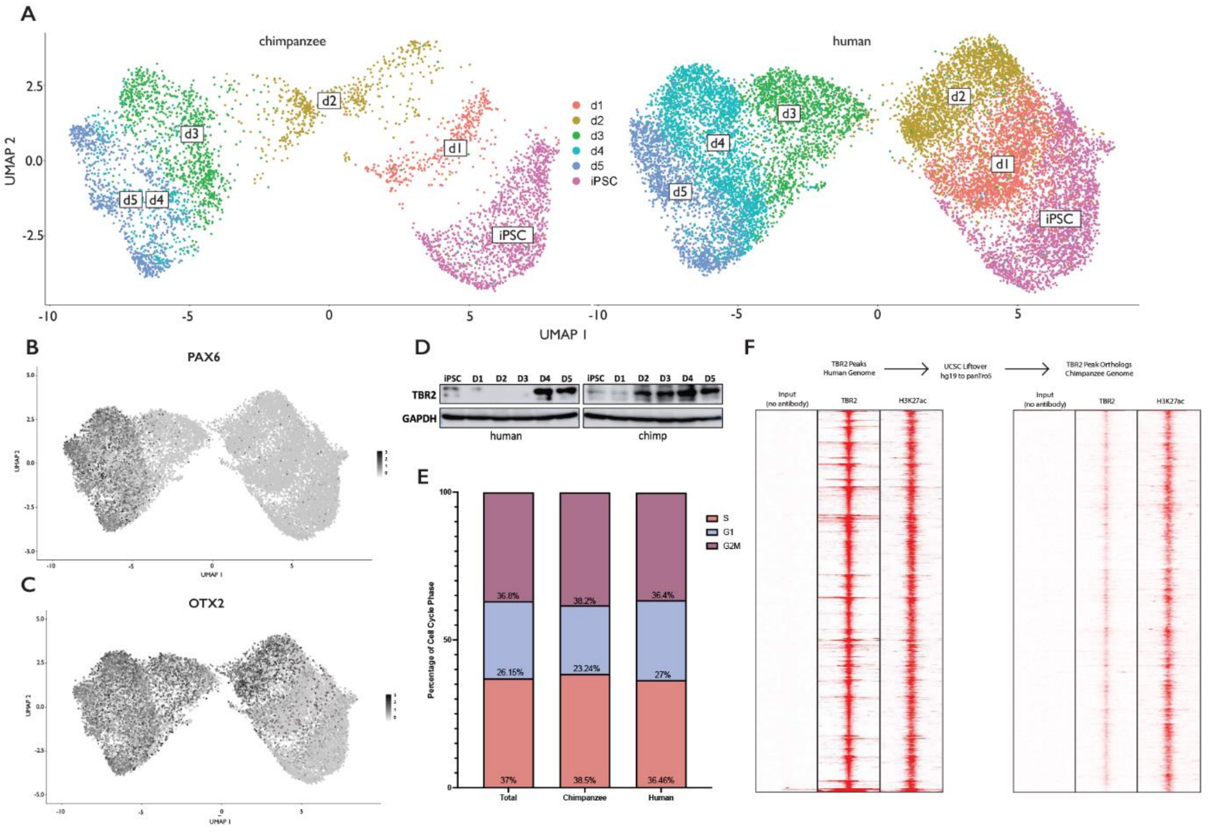


---

<u>Second decision letter</u>

MS ID#: DEVELOP/2021/200413

MS TITLE: Young transposable elements rewired gene regulatory networks in human and chimpanzee hippocampal intermediate progenitors

AUTHORS: Sruti Patoori, Samantha Barnada, Christopher Large, John Murray, and MARCO TRIZZINO
ARTICLE TYPE: Research Article

© 2022. Published by The Company of Biologists under the terms of the Creative Commons Attribution License (https://creativecommons.org/licenses/by/4.0/). 

I am happy to tell you that your manuscript has been accepted for publication in Development, pending our standard ethics checks.

Reviewer 2

*Advance summary and potential significance to field*

as in my first review

*Comments for the author*

The authors added substantial data to this revised version of the MS. In particular, the single cell RNAseq data add information about culture composition that was required. I am satisfied with this revisions and I consider the MS acceptable for publication.

© 2022. Published by The Company of Biologists under the terms of the Creative Commons Attribution License (https://creativecommons.org/licenses/by/4.0/).
